# Supplementary material for: Elucidating Sequence and Structural Determinants of Carbohydrate Esterases for Complete Deacetylation of Substituted Xylans
Source: Molecules. 2022 Apr 20;27(9):2655. doi: 10.3390/molecules27092655 (PMC9105624; doi:10.3390/molecules27092655)
Supplement: Supplementary file 1 [file molecules-27-02655-s001.zip › molecules-1617392-supplementary.pdf]

Elucidating sequence and structural determinants of complete deacetylation of substituted xylans

Leena Penttinen [1], Vera Kouhi [2], Régis Fauré [3], Tatiana Skarina[4], Peter Stogios [4], Emma Master [4,5], and Edita Jurak [6,*]

Supplementary material

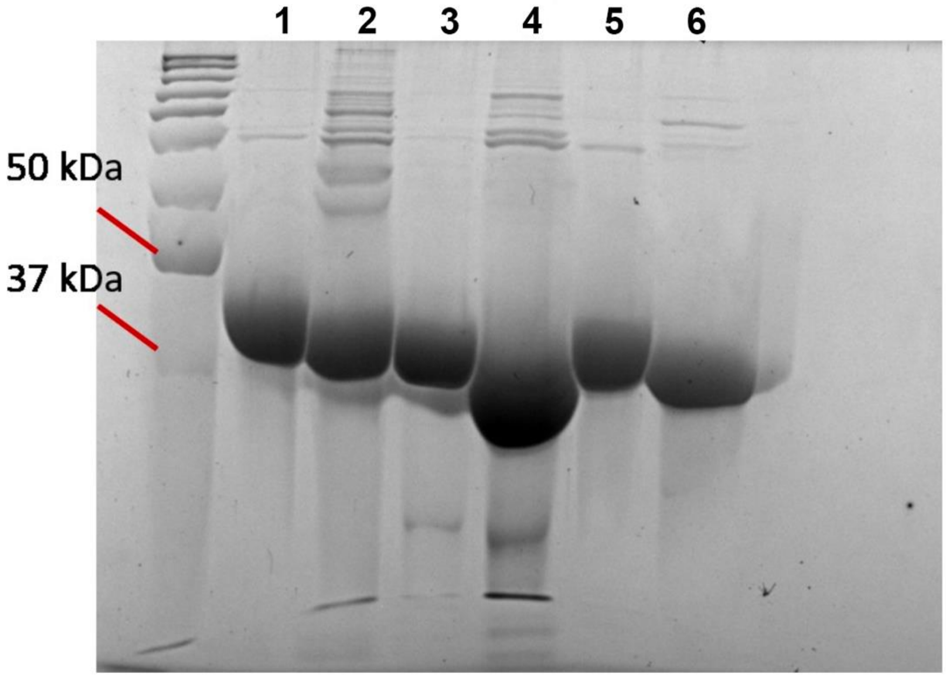

Figure S1. Studied acetyl xylan esterases (AcXE) on SDS-PAGE. Sample 1: *Fsp*AcXE, 2: *Fsp*F52AcXE, 3: *Csp*AcXE, 4: *Pbe*AcXE, 5: *Fre*AcXE, 6: *Aim*AcXE.

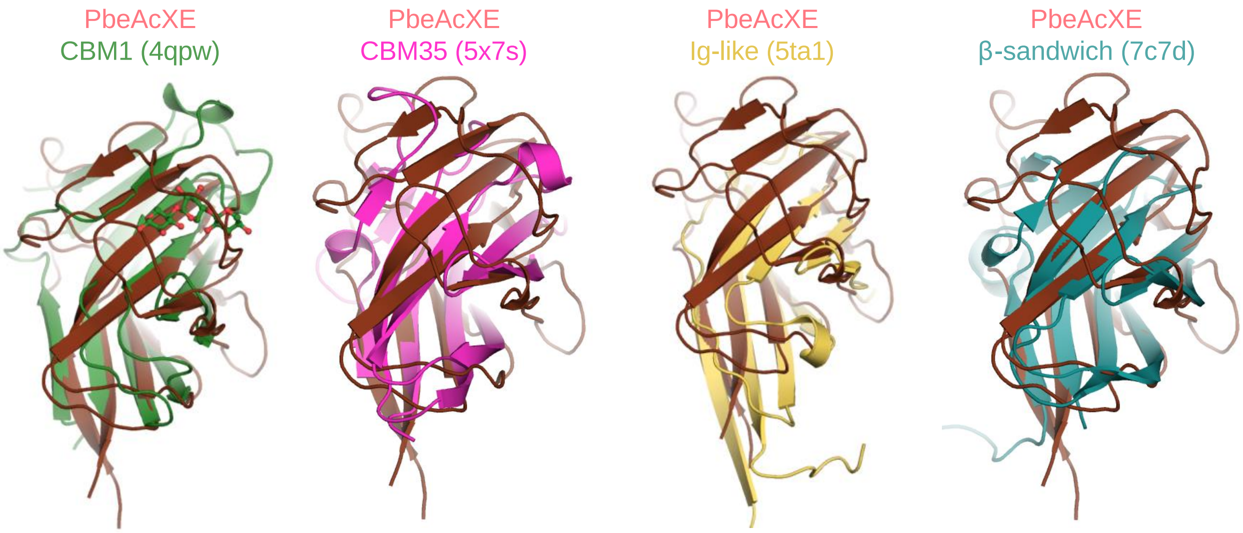

Figure S2. A crystal structure of the CBM domain of *Pbe*AcXE superimposed with a CBM1 domain from GH10 enzyme from *Bacteroides intestinalis* (PDB ID 4qpw), CBM35 from a GH31 enzyme from *Paenibacillus sp. 598K* (PDB ID 5x7s), and the Ig-like domain from a GH86 enzyme from *Bacteroides uniformis* (PDB ID 5ta1) and the β-sandwich domain from a GH87 enzyme from *Streptomyces thermodiastaticus* (PDB ID 7c7d).

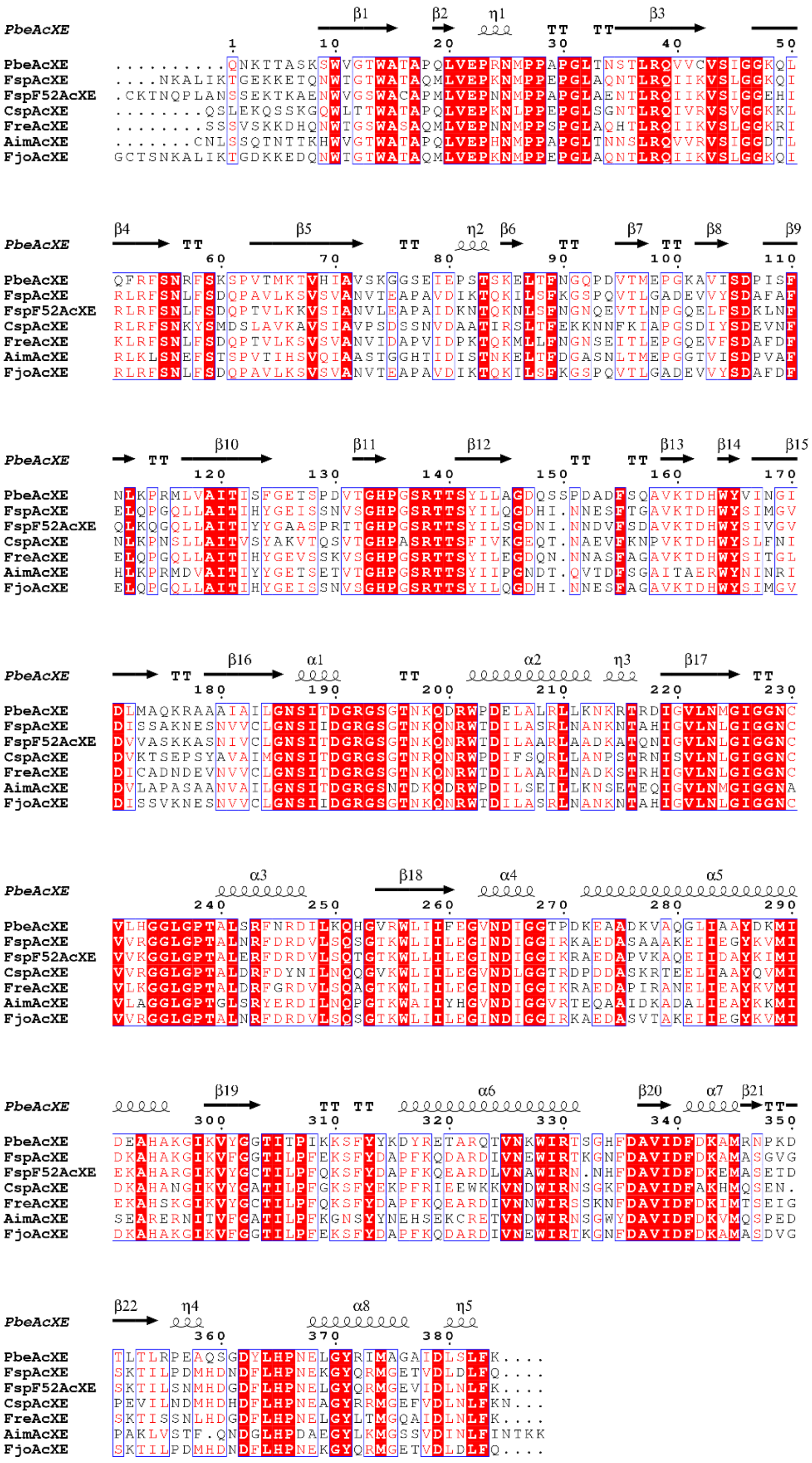

Figure S3. Sequence alignment of the studied acetyl xylan esterases (AcXE) and AcXE from *Flavobacterium johnsoniae* UW101 (*Fjo*AcXE). Figure is prepared with ESPript. 3.0 server.

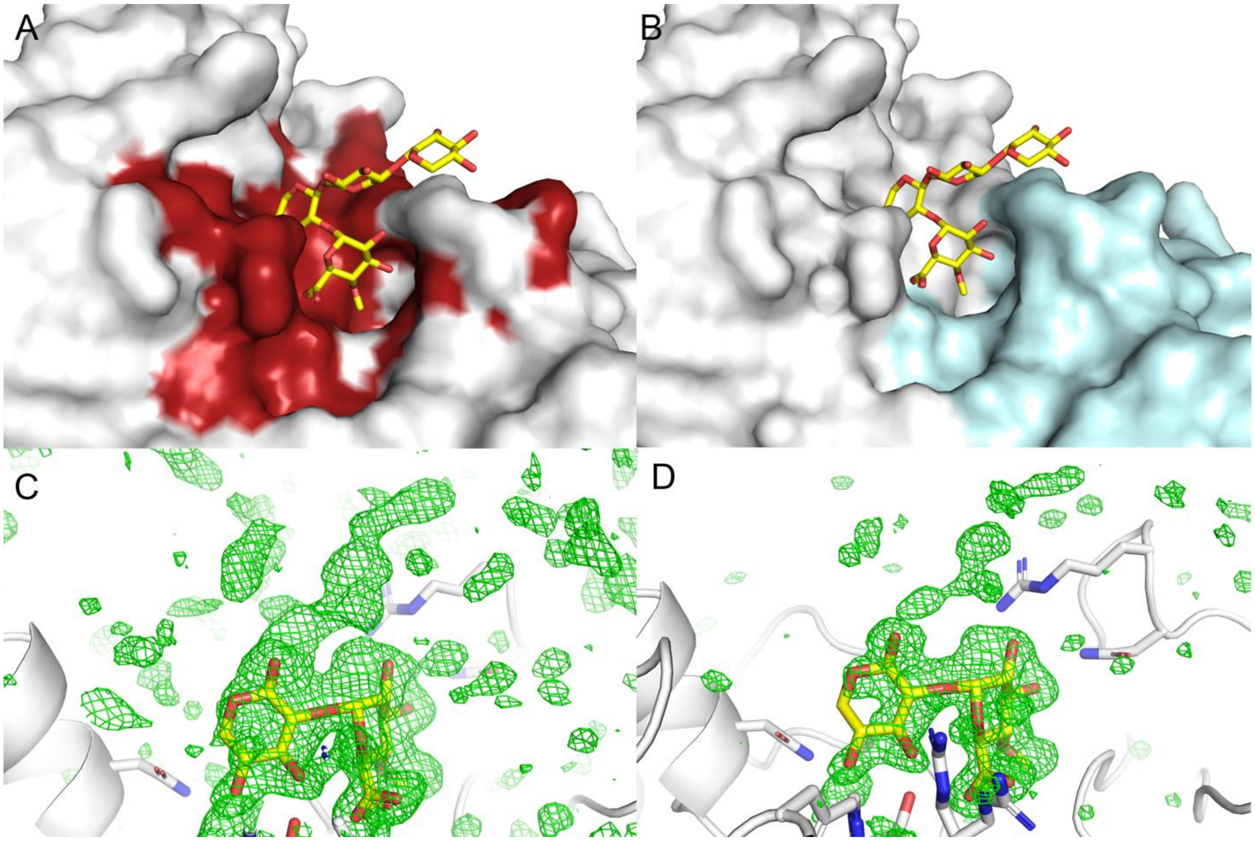

Figure S4. Surface representation of a complex structure of *Prolixibacter bellariivorans* acetyl xylan esterase with $U^{4m2}XX$, MeGlc*p*A-(1→2)-Xyl*p*(1→4)-Xyl*p*-(1→4)-Xyl*p*. A) The conserved residues near the active site are shown in red. B) The catalytic SGHN domain is shown in grey and the CBM domain in pale blue. C) Positive $F_o–F_c$ electron density in the complex structure after removing the ligand is shown at 2σ contour level in green. D) The same positive $F_o–F_c$ electron density is shown at 1σ contour level.

Table S1. The six selected *Fjo*AcXE-homologues.

PULDB ID	K392DRAFT_2214	FF52_18088	EW79DRAFT_2436	T426DRAFT_00687	WP_035683315.1	AlkimDRAFT_0871
Protein name	*Fsp*AcXE	*Fsp*F52AcXE	*Csp*AcXE	*Pbe*AcXE	*Fre*AcXE	*Aim*AcXE
GenBank ID	WP_073411463.1	WP_008467835.1.	WP_047423182.1	WP_027586161.1	WP_035683315.1	WP_044117739.1
Species	*Flavobacterium* sp. 40S8	*Flavobacterium* sp. F52	*Chryseobacterium* sp. YR480	*Prolixibacter bellariivorans*	*Flavobacterium reichenbachii*	*Alkaliflexus imshenetskii* DSM 15055 AlkimDRAFT_0871
Identity with FjoAcXE, %	98.1	69.4	53.3	51.6	73.9	47
Molecular mass, kDa	42.3	42.8	42.8	41.9	42.0	42.2
Theoretical pI	6.7	6.7	9.0	9.4	6.3	5.3
Signal sequence	+	+	+	+	+	+
Yield (mg)	40	9.2	12	8	8.3	14

Molecular mass and theoretical pI were calculated with ProtParam Tool (http://web.expasy.org/protparam/).

Table S2. Substrates used in this study.

Substrate name	IUPAC name	Producer	Activity detection method
Wheat arabinoxylan (medium viscosity)	Arabinoxylan (1,2;1,3-α-Araf)	Megazyme	PAHBAH assay
Arabinoglucuronoxylan (oat spelt water soluble part separated after boiling)	Arabinoglucuronoxylan	Sigma-Aldrich	PAHBAH assay
CM-Cellulose 4M (medium viscosity)	Cellulose	Megazyme	PAHBAH assay
Galacto-xyloglucan (Tamarind, high viscosity)	Galacto-xyloglucan	Megazyme	PAHBAH assay
Lichenan (Icelandic moss)	Glucan (1,3; 1,4-β-D-)	Megazyme	PAHBAH assay
Pullulan	Glucan (1,4; 1,6-α-D-)	Megazyme	PAHBAH assay
Dextran	Glucan (1,6-α-D-)	Megazyme	PAHBAH assay
Glucomannan Konjac (low viscosity)	Glucomannan, acetylated	Megazyme	PAHBAH assay
Mannan	Mannan (1,4-β-D-)	Megazyme	PAHBAH assay
Arabinan (sugar beet)	Pectin (1,5-α-L-Araf)	Megazyme	PAHBAH assay
Arabinogalactan (Larch wood)	Pectin (1,3;1,4;1,6-β-D-Gal; 1,4;1,6-α-L-Araf)	Megazyme	PAHBAH assay
Pectic galactan (Lupin)	Pectin (Galactan (1,4-β-D))	Megazyme	PAHBAH assay

Galactan (Lupin)	Pectin (Galactan (1,4-β-D-)); Arabinofuranosidase treated lupin pectic galactan)	Megazyme	PAHBAH assay
Rhamnogalacturonan (soy bean)	Rhamnogalacturon	Megazyme	PAHBAH assay
Rhamnogalacturonan I (potato)	Rhamnogalacturonan I	Megazyme	PAHBAH assay
Glucuronoxylan	Glucuronoxylan	Megazyme	PAHBAH assay
*p*NP-arabinofuranoside	4-Nitrophenyl α-L-arabinofuranoside	Sigma-Aldrich Inc., USA	*p*NP-assay
*p*NP-glucopyranoside	4-Nitrophenyl β-D-glucopyranoside	Sigma-Aldrich Inc., USA	*p*NP-assay
*p*NP-mannopyranoside	4-Nitrophenyl β-D-mannopyranoside	Sigma-Aldrich Inc., USA	*p*NP-assay
*p*NP-xylopyranoside	4-Nitrophenyl β-D-xylopyranoside	Sigma-Aldrich Inc., USA	*p*NP-assay
*p*NP-chitobioside	4-Nitrophenyl *N,N'*-diacetyl-β-chitobioside	Sigma-Aldrich Inc., USA	*p*NP-assay
*p*NP-α-galactopyranoside	4-Nitrophenyl α-D-galactopyranoside	Sigma-Aldrich Inc., USA	*p*NP-assay
*p*NP-fucopyranoside	4-Nitrophenyl- α-L-fucopyranoside	Sigma-Aldrich Inc., USA	*p*NP-assay
*p*NP-β-galactopyranoside	4-Nitrophenyl β-D-galactopyranoside	Sigma-Aldrich Inc., USA	*p*NP-assay
*p*NP-acetate	4-Nitrophenyl acetate	Sigma-Aldrich Inc., USA	*p*NP-assay,

			Minimal enzyme concentration assay; pH optimum assay
4-MU-acetate	4-Methylumbelliferyl acetate	Sigma-Aldrich Inc., USA	4-MU-acetate-assay, Minimal enzyme concentration assay; pH optimum assay
Acetylated and feruloylated corn xylooligosaccharade (CF-XOS)		Mild acid hydrolysis of corn fiber, received as a gift from prof. Mirjam Kabel (University of Wageningen, The Netherlands)	Secondary screen
Acetylated glucurono-xylooligosaccharides		steam extraction of milled chips from Eucalyptus wood, received as a gift from prof. Maija Tenkanen (University of Helsinki, Finland)	Secondary screen
Xylooligosaccharide mixture	Per-*O*-acetylated xylo-oligosaccharides (DP 4-7)	TBI, France	Secondary screen

Table S3. The pH optimum of *Fjo*AcXE homologs was evaluated by measuring enzyme activity on ?? mM 4-MUA after 30 minutes at 40°C between pH 3.5 – 9.0.

	pH 3.5	pH 4.5	pH 5.5	pH 6.0	pH 6.5	pH 7.0	pH 7.5	pH 8.0	pH 9.0
*Fsp*AcXE	4%	26%	63%	94%	100%	93%	87%	44%	6%
*Fsp*F52AcXE	13%	45%	52%	86%	85%	72%	100%	89%	0%
*Csp*AcXE	2%	13%	37%	59%	100%	85%	70%	50%	0%
*Pbe*AcXE	26%	42%	67%	86%	54%	100%	88%	34%	0%
*Fre*AcXE	4%	37%	85%	88%	89%	100%	96%	52%	0%
*Aim*AcXE	9%	46%	86%	83%	65%	87%	100%	85%	39%

Table S4. The temperature optimum of *Fjo*AcXE homologs was evaluated by measuring enzyme activity on ?? mM 4-MUA after 24 hours at 20, 40, 55 and 70°C and the optimum pH of the enzyme.

20°C	0 min	30 min	1 h	2 h	4 h	6 h	24 h
*Fsp*AcXE	81 %	100 %	77 %	65 %	70 %	98 %	24 %
*Fsp*F52AcXE	91 %	100 %	78 %	55 %	55 %	67 %	32 %
*Csp*AcXE	100 %	83 %	109 %	100 %	111 %	66 %	96 %
*Pbe*AcXE	97 %	87 %	97 %	94 %	66 %	95 %	100 %
*Fre*AcXE	82 %	100 %	100 %	71 %	58 %	48 %	41 %
*Aim*AcXE	100 %	95 %	66 %	72 %	40 %	30 %	7 %
40°C	0 min	30 min	1 h	2 h	4 h	6 h	24 h
*Fsp*AcXE	77 %	100 %	59 %	64 %	55 %	45 %	5 %
*Fsp*F52AcXE	82 %	77 %	62 %	100 %	45 %	42 %	14 %
*Csp*AcXE	96 %	100 %	67 %	91 %	68 %	66 %	70 %
*Pbe*AcXE	78 %	75 %	68 %	62 %	100 %	41 %	57 %
*Fre*AcXE	95 %	76 %	88 %	68 %	100 %	29 %	44 %
*Aim*AcXE	100 %	77 %	62 %	32 %	14 %	8 %	0 %

55°C	0 min	30 min	1 h	2 h	4 h	6 h	24 h
*Fsp*AcXE	100 %	63 %	24 %	15 %	4 %	3 %	1 %
*Fsp*F52AcXE	100 %	71 %	68 %	69 %	43 %	37 %	8 %
*Csp*AcXE	100 %	85 %	64 %	49 %	31 %	32 %	4 %
*Pbe*AcXE	100 %	85 %	83 %	91 %	60 %	57 %	29 %
*Fre*AcXE	100 %	67 %	50 %	15 %	12 %	10 %	2 %
*Aim*AcXE	100 %	27 %	22 %	1 %	0 %	1 %	1 %
70°C	0 min	30 min	1 h	2 h	4 h	6 h	24 h
*Fsp*AcXE	100 %	0 %	0 %	0 %	0 %	0 %	0 %
*Fsp*F52AcXE	100 %	0 %	0 %	0 %	0 %	0 %	0 %
*Csp*AcXE	100 %	0 %	0 %	0 %	0 %	0 %	0 %
*Pbe*AcXE	100 %	0 %	0 %	0 %	0 %	0 %	0 %
*Fre*AcXE	100 %	0 %	0 %	0 %	0 %	0 %	0 %
*Aim*AcXE	100 %	0 %	0 %	0 %	0 %	0 %	0 %

Table S5. X-ray crystallographic statistics.

Structure	PbeAcXE	PbeAcXE MeGlcpA-Xylp complex	PbeAcXE acetate complex	CspAcXE	FjoAcXE CBM
**PDB code**	7TOG	7TOH	7TOI	7TOJ	7TOK
**Data collection**					
Space group	$P2_1$	$P2_1$	$P2_1$	C2	$C222_1$
Cell dimensions					
a, *b*, *c* (Å)	57.7, 42.6, 76.8	57.6, 42.8, 76.7	57.4, 42.6, 74.5	120.2, 77.3, 42.1	60.1, 120.1, 86.6
α, β, γ, (°)	90, 108.3, 90	90, 108.4, 90	90, 108.3, 90	90, 96.9, 90	90, 90, 90
Resolution, Å	28.78 – 1.35	28.53 – 1.26	54.49 – 1.13	36.33 – 1.30	60.48 – 2.45
R_{merge}[a]	0.106 (1.282)*	0.104 (1.467)	0.116 (0.717)	0.067 (1.598)	0.054 (1.55)
R_{pim}[b]	0.035 (0.515)	0.030 (0.583)	0.023 (0.349)	0.022 (0.648)	0.026 (0.745)
$CC_{1/2}$	0.997 (0.562)	0.998 (0.557)	0.999 (0.657)	0.999 (0.536)	0.999 (0.519)
$I / \sigma(I)$	11.7 (1.4)	13.6 (1.2)	17.5 (2.2)	15.0 (1.2)	18.6 (0.8)
Completeness, %	99.5 (98.7)	97.4 (92.4)	98.1 (91.7)	99.7 (99.5)	99.9 (100)
Multiplicity	8.9 (7.0)	11.8 (7.2)	20.2 (5.1)	9.4 (7.0)	4.8 (4.9)
**Refinement**					
Resolution, Å	28.78 –1.35	28.53 – 1.26	54.45 – 1.13	35.36 – 1.30	43.32 – 2.45
No. unique reflections: working, test	77700, 3868	93456, 4701	128849, 1997	93351, 4615	11916, 560
R_{work}/R_{free}[c]	15.0/18.0 (29.7/27.8)	13.5/16.9 (26.0/28.4)	15.3/16.5 (25.1/28.9)	14.0/16.4 (29.5/32.4)	23.0/28.4 (40.0/45.3)
No. atoms					
Protein	2955	2964	5962	2861	1979
Ligand	N/A	23	7	N/A	N/A
Solvent	N/A	N/A	N/A	3	N/A
Water	656	726	708	591	10
B-factors					
Protein	21.9	20.2	17.8	26.0	99.6
Ligand	N/A	26.8	16.1	N/A	N/A
Solvent	N/A	N/A	N/A	20.7	N/A
Water	36.6	36.7	30.8	41.1	75.3
R.m.s. deviations					
Bond lengths, Å	0.017	0.012	0.013	0.008	0.004
Bond angles, °	1.547	1.373	1.280	1.072	0.966
Ramachandran plot					
Favored, %	96.5	97.6	97.1	97.2	88.7
Allowed, %	3.5	2.4	2.9	2.5	10.5
Outliers, %	0	0	0	0.3	0.8

*All values in brackets refer to values in highest resolution shells.

[a]$R_{merge} = \Sigma_{hkl}\Sigma_j|I_{hkl,j} - \langle I_{hkl}\rangle|/\Sigma_{hkl}\Sigma_j I_{hk,j}$, where $I_{hkl,j}$ and $\langle I_{hkl}\rangle$ are the *j*th and mean measurement of the intensity of reflection *j*.

[b]$R_{pim} = \Sigma_{hkl}\sqrt{(n/n-1)}\ \Sigma^n_{j=1}|I_{hkl,j} - \langle I_{hkl}\rangle|/\Sigma_{hkl}\Sigma_j I_{hk,j}$

[c]R_{work} or $R_{free} = \Sigma|F_p^{obs} - F_p^{calc}|/\Sigma F_p^{obs}$, where F_p^{obs} and F_p^{calc} are the observed and calculated structure factor amplitudes, respectively.

N/A = not applicable.

Table S6. Structural orthologs of PbeAcXE and CspAcXE from Dali search (full-length structures).

PbeAcXE orthologs					
**PDB code**	**Protein name**	**Known or putative function**	**Z-score**	**RMSD (Å over X Cα atoms)**	**Sequence identity %**
4rsh	putative lipolytic protein of G-D-S-L family from *Desulfitobacterium hafniense DCB-2*	Unknown	22.3	1.9, 172	21
4rw0	lipolytic protein G-D-S-L family from *Veillonella parvula DSM 2008*	Unknown	20.9	2.2, 173	24
4jgg	TesA from *Pseudomonas aeruginosa*	Thioesterase, lysophospholipase	20.8	2.2, 175	21
4ppy	putative acylhydrolase (BF3764) from *Bacteroides fragilis NCTC 9343*	Unknown	20.2	2.7, 191	22
6nkd	Lipase Lip_vut3 from Goat Rumen metagenome.	Lipase	20.1	2.5, 186	16
1yzf	lipase/acylhydrolase from *Enterococcus faecalis*	Unknown	20.0	2.5, 182	22
7ddy	acetyl xylan esterase AlAXEase from *Arcticibacterium luteifluviistationis*	acetylxylan esterase	19.9	2.5, 177	23
5tic	*E. coli* Acyl-CoA thioesterase I / TesA	Thioesterase	19.5	2.4, 171	20
4jko	Axe2 (Axe2_S15A), an acetylxylan esterase from *Geobacillus stearothermophilus*	acetylxylan esterase	19.2	2.7, 178	20
5b5s	acetyl esterase CE3 from *Talaromyces cellulolyticus*	CE3 acetyl esterase	18.2	2.1, 167	19
6uqz	ChoE from *Pseudomonas aeruginosa PAO1*	Acetylcholinesterase	17.4	2.8, 186	15
2waa	CtCE2 from *Cellvibrio japonicus*	CE2 acetyl esterase	15.0	3.3, 194	15
3u37	Est2A from *Butyrivibrio proteoclasticus B316*	CE2 acetyl esterase	14.2	3.8, 205	13
**CspAcXE orthologs**					
4rsh	putative lipolytic protein of G-D-S-L family from *Desulfitobacterium hafniense DCB-2*	Unknown	19.5	1.8, 156	28
1vjg	gdsl-like lipase (alr1529) from *nostoc sp. pcc 7120*	Unknown	19.4	2.5, 178	15
7ddy	acetyl xylan esterase AlAXEase from *Arcticibacterium luteifluviistationis*	acetylxylan esterase	18.6	2.5, 166	21
5tic	*E. coli* Acyl-CoA thioesterase I / TesA	Thioesterase	18.6	2.5, 166	16

4rw0	lipolytic protein G-D-S-L family from *Veillonella parvula DSM 2008*	Unknown	18.4	2.2, 162	20
4jgg	TesA from *Pseudomonas aeruginosa*	Thioesterase, lysophospholipase	18.4	2.4, 163	22
4jko	Axe2 (Axe2_S15A), an acetylxylan esterase from *Geobacillus stearothermophilus*	acetylxylan esterase	17.7	2.7, 165	17
4ppy	putative acylhydrolase (BF3764) from *Bacteroides fragilis NCTC 9343*	Unknown	16.8	2.7, 168	23
5b5s	acetyl esterase CE3 from *Talaromyces cellulolyticus*	CE3 acetyl esterase	16.5	2.3, 160	18
6uqz	ChoE from *Pseudomonas aeruginosa PAO1*	Acetylcholinesterase	15.7	2.6, 172	12
2waa	CtCE2 from *Cellvibrio japonicus*	CE2 acetyl esterase	15.0	3.7, 193	11
3u37	Est2A from *Butyrivibrio proteoclasticus B316*	CE2 acetyl esterase	13.4	3.6, 187	14
